# Solving the Manufacturing Cell Design Problem through Binary Cat Swarm Optimization with Dynamic Mixture Ratios

**DOI:** 10.1155/2019/4787856

**Published:** 2019-02-14

**Authors:** Ricardo Soto, Broderick Crawford, Angelo Aste Toledo, Hanns de la Fuente-Mella, Carlos Castro, Fernando Paredes, Rodrigo Olivares

**Affiliations:** ^1^Pontificia Universidad Católica de Valparaíso, Avenida Brasil 2241, Valparaíso 2362807, Chile; ^2^Universidad Técnica Federico Santa María, Avenida España 1680, Valparaíso 2390123, Chile; ^3^Universidad Diego Portales, Av. Ejército 441, Santiago 8370109, Chile; ^4^Universidad de Valparaíso, General Cruz 222, Valparaíso 2603631, Chile

## Abstract

In this research, we present a Binary Cat Swarm Optimization for solving the Manufacturing Cell Design Problem (MCDP). This problem divides an industrial production plant into a certain number of cells. Each cell contains machines with similar types of processes or part families. The goal is to identify a cell organization in such a way that the transportation of the different parts between cells is minimized. The organization of these cells is performed through Cat Swarm Optimization, which is a recent swarm metaheuristic technique based on the behavior of cats. In that technique, cats have two modes of behavior: seeking mode and tracing mode, selected from a mixture ratio. For experimental purposes, a version of the Autonomous Search algorithm was developed with dynamic mixture ratios. The experimental results for both normal Binary Cat Swarm Optimization (BCSO) and Autonomous Search BCSO reach all global optimums, both for a set of 90 instances with known optima, and for a set of 35 new instances with 13 known optima.

## 1. Introduction

Group technology is a manufacturing philosophy in which similar parts are identified and grouped together to take advantage of their similarities in design and production [1] by organizing similar parts into part families, where each part of the family has similar design and manufacturing characteristics. The basic concept of group technology has been practiced for many years around the world, as part of good engineering and scientific management practices [2, 3], which states that similar things should be manufactured in a similar way [4].

The Manufacturing Cell Design Problem (MCDP) is an application of group technology to organize cells containing a set of machines to process a family of parts [5]. In this context, MCDP involves the creation of an optimal design of production plants, in which the main objective is to minimize the movement and exchange of material between these cells, thus generating greater productivity and reducing production costs.

The Manufacturing Cell Design Problem belongs to the complex NP-hard class of problems, and then exploring good search algorithms is always a challenging task from the optimization and now also from the artificial intelligence world [5]. In particular, in this paper, an efficient metaheuristic implementation is proposed to tackle this problem, demonstrating through several benchmark instances its performance (various global optima are reached), which is also valuable from an artificial intelligence and optimization standpoint. Additionally, this algorithm includes an Autonomous Search Component (dynamic mixture ratio), which is currently an important research trend in the optimization and metaheuristic sphere. Metaheuristics are intrinsically complex to be configured in order to reach good results, and Autonomous Search comes to facilitate this task by letting the metaheuristic itself to self-tune its internal configuration without the need of a user expert for reaching good results. To the best of our knowledge, the work done on Autonomous Search in metaheuristics is very recent, and no Autonomous Search work for cat swarm exists.

The research work that has been done to solve the problem of cell formation has followed two complementary lines, which can be organized into two groups: approximate methods and exact methods. Approximate methods are mostly focused on finding an optimal solution in a limited time; however, they do not guarantee a global optimum. Exact methods, on the contrary, aim to fully analyze the search space to ensure a global optimum [6]; however, these algorithms are quite time-consuming and can only solve cases of very limited size. For this reason, many research efforts have focused on the development of heuristics, which find near-optimal solutions within a reasonable period of time.

This research focuses on solving the MCDP through a recent metaheuristic in the vein of Swarm Intelligence (SI) [7] called Binary Cat Swarm Optimization (BCSO) [8]. This algorithm was generated from observations of cat behavior in nature, in which cats either hunt or remain alert. BCSO is based on the CSO algorithm, recently proposed by Chu and Tsai [9]. The difference is that in BCSO, the vector position consists of ones and zeros, instead of real numbers (CSO), and the proposed alternate version makes use of a dynamic mixture ratio.

As aforementioned, reaching good results for problems belonging from the NP class is always a challenging and appealing task from the optimization and artificial intelligence world. In this research, our goal was to provide an intelligent algorithm for solving this problem by additionally integrating self-tuning features, which is a very recent research trend in the optimization and metaheuristic sphere.

## 2. Theoretical Framework

The formation of manufacturing cells has been researched for many years. One of the first investigations focused on resolving this set of problems was Burbidge's work in 1963 [4], which proposed the use of an incidence matrix reorganized into a Block Diagonal Form (BDF) [4]. In recent years, many exact and heuristic algorithms have been proposed in the literature to solve MCDP. Such metaheuristic techniques include genetic Algorithm (GA) [10], inspired by biological evolution and its genetic-molecular basis; the Neural Network (NN) [11] that takes the behavior of neurons and the connections of the human brain; and Constraint Programming (CP) [12] where the relationships between the variables are expressed as constraints. For extensive reviews of previous research and other methods of cell formation, see Selim et al. [1].

Among the metaheuristics used for cell formation, there is also the branch of Swarm Intelligence, which was initially introduced by Beni and Wang in 1989 [13]. Inspired by nature, Swarm Intelligence systems are typically formed by a population of simple agents who interact locally with each other and with their environment and who are able to optimize an overall objective through the search for collaboration in a space [14]. Within this branch, the main techniques are Particle Swarm Optimization (PSO) designed and presented by Eberhart et al. [7, 9] in 1995; Ant Colony Optimization (ACO), which is a family of algorithms derived from Dorigo's 1991 work based on the social behavior of ants [15, 16]; Migrating Birds Optimization (MBO) [17] algorithm based on the alignment of migratory birds during flight; Artificial Fish Swarm Algorithm (AFSA) [18], based on the behavior of fish to find food by themselves or by following other fish; and the discrete Cat Swarm optimization (CSO) Technique presented in 2007 by Chu and Tsai [9], which is based on the behavior of cats. Interestingly, the CSO cat corresponds to a particle in PSO, with a small difference in its algorithms [19, 20]. CSO and PSO were originally developed for continuous value spaces, but there are a number of optimization problems where the values are discrete [21].

## 3. The Manufacturing Cell Design Problem

The Manufacturing Cell Design Problem (MCDP) divides an industrial production plant into a number of cells. Each cell contains machines with similar process types or part families, determined according to the similarity between parts [4]. A manufacturing cell can be defined as an independent group of functionally different machines, located together, dedicated to the manufacture of a family of similar parts. In addition, a family of parts can be defined as a collection of parts that are similar, either because of their geometric shape and size or because similar processing steps are required to manufacture them [22].

The goal of MCDP is to identify a cell organization in a way that minimizes the transport of different parts between cells, in order to reduce production costs and increase productivity. The idea is to represent the processing requirements of machine parts through an incidence matrix called machine part. This reorganization involves the formulation of two new matrices called machine-cell and part-cell.

A detailed mathematical definition of the formulation of the machine-part clustering problem is defined by the optimization model explained below [6]:

(i) *M:* number of machines
(ii) *P:* number of parts
(iii) *C*: number of cells
(iv) *i*: machine index (*i*=1,2,…, *M*)
(v) *j*: part index (*j*=1,2,…, *P*)
(vi) *k*: cell index (*k*=1,2,…, *C*)
(vii) *M*_max_: maximum number of machines per cell
(viii) *A*=[*a*_*ij*_]: machine-to-part binary incidence matrix, where
  (1) $$\begin{array}{c} a_{ij}=\left\{\begin{array}{cc} 1, & \text{if machine }i\text{ processes a part }j,\\ 0, & \text{otherwise}. \end{array}\right. \end{array}$$
(ix) *B*=[*b*_*ij*_]: machine-to-part binary incidence matrix, where
  (2) $$\begin{array}{c} b_{ik}=\left\{\begin{array}{cc} 1, & \text{if machine }i\text{ belongs to cell }k,\\ 0, & \text{otherwise}. \end{array}\right. \end{array}$$
(x) *C*=[*c*_*jk*_]: machine-to-part binary incidence matrix, where
  (3) cjk=1,if part j belongs to cell k,0,otherwise.

## 4. Binary Cat Swarm Optimization

There are about thirty different species of known felines, e.g., lions, tigers, leopards, common housecat, etc. [23]. Although they have different living environments, cats share similar behavioral patterns [24]. For wild cats, the ability to hunt ensures food supply and survival of the species [25]. To hunt their food, wild cats form groups ranging from 2–15 individuals [26]. Domestic cats also show the same ability to hunt and are curious about moving objects [26–28]. Although cats might seem to be resting most of the time, even when awake [29, 30], they are actually in a constant state of alert; without moving, they may be listening or have their eyes open to look around [31]. BCSO [8] was formulated on the basis of all these behaviors and is an optimization algorithm that mimics the natural behavior of cats [9, 32, 33]. The authors identified two main modes of behavior for simulating cats [3, 34–39]:

- Seeking mode: exploration-oriented mode, where cats are attracted by moving objects and have a high hunting capacity. Cats may seem to spend most of their time resting, but in fact, they are constantly alert when moving slowly.
- Tracing mode: exploitation-oriented mode, where cats detect a prey and run after it, spending a lot of energy due to its rapid movements. In this way, the cats follow the best in their group.

In BCSO, these two behaviors are mathematically modeled to solve complex optimization problems. The first decision is to define the number of cats needed for each iteration. Each cat, represented by cat_*k*_, where *k* ∈ {1,2,…, *C*}, has its own position consisting of *M* dimensions composed of ones and zeros (1 and 0). In addition, they have speed for each dimension *d*, a flag to indicate whether the cat is in the seeking or tracing mode, and finally a fitness value that is calculated based on the MCDP. The BCSO keeps looking for the best solution until iterations are finalized. In BCSO, each cat_*x*_ represents a MCDP solution through a machine-cell matrix, where *x* identifies the cat and *d* are the position bits of the cat. In addition, the constraint matrix ensures that each row *i* is covered by at least one column.

Algorithm 1 describes the general BCSO pseudocode where the mixture ratio (MR) is a percentage that determines the number of cats in the seeking mode.

### 4.1. Seeking Mode

This submodels the state of the cat, which is resting, looking around, and seeking the next position to move towards. The seeking mode has the following essential factors:

- PMO: probability of mutation operation, a percentage that defines the mutation probability for the selected dimension.
- CDC: counts of dimensions to change, a percentage that indicates how many dimensions are candidates to change.
- SMP: seeking memory pool, a positive integer used to define the memory size for each cat. SMP indicates the points to be scanned by the cat and can be different for different cats.

The following pseudocode describes the behavior of the cat in the seeking mode. Here, FS_*i*_ is the fitness of the *i*th cat, and FS_*b*_=FS_max_ finds the minimum solution and FS_*b*_=FS_min_ the maximum solution. To solve the MCDP, we use FS_*b*_=FS_max_.

Step 1: create SMP copies of current cat_*x*_.
Step 2: for each copy:
for dimensions that are candidates for change (based on CDC percentage):
get a random number (rand) between 0 and 1
if rand < PMO, then the position changes.
Step 3: evaluate Fitness of all copies.
Step 4: calculate the selection probability by applying a roulette wheel or, by default, choose the best copy according to Fitness.
  (4) $$\begin{array}{c} P_{i}=\left|\frac{{\text{FS}}_{i}-{\text{FS}}_{b}}{{\text{FS}}_{\text{max}}{\;-\text{ FS}}_{\text{min}}}\right|. \end{array}$$
Step 5: evaluate if the chosen copy is a better solution than the currently selected cat, and replace accordingly.

**Figure 1:**
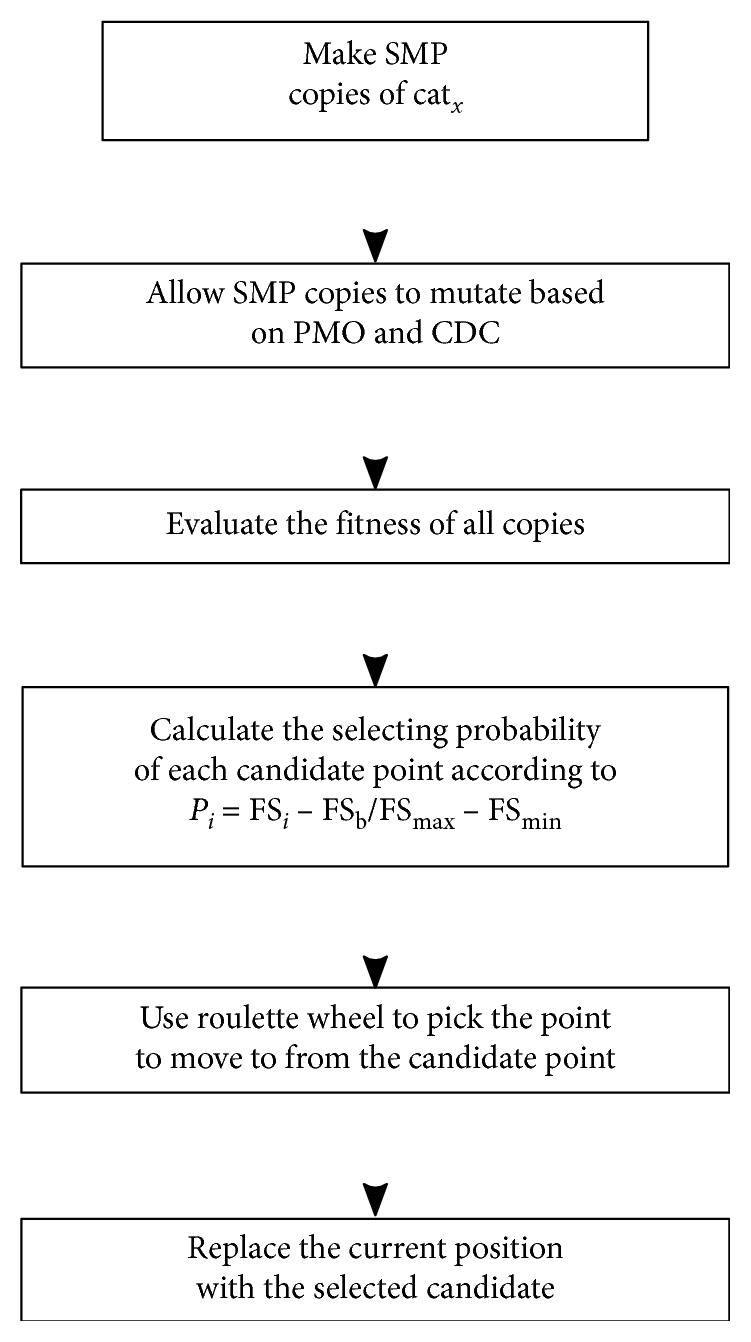
Seeking mode.

Figure 1 shows the flow chart of the behavior of the cat in the seeking mode.

### 4.2. Tracing Mode

This submodel is used to model the state of the cat in hunting or tracing behavior, where the cats are moving towards the best solution obtained so far. Once a cat enters the tracing mode, it moves according to its own velocities for each dimension. Each cat has two velocity vectors, defined as *V*_kd_^1^ and *V*_kd_^0^, where *V*_kd_^0^ is the probability that the bits of the cat change to zero and *V*_kd_^1^ is the probability they change to one. The velocity vector changes its meaning with the probability of mutation for each dimension *d*. The tracing mode action is described in the following pseudocode.

Step 1: calculate *d*_kd_^1^ and *d*_kd_^0^ according to the following expression, where *X*_best,*d*_ is the dimension *d* of the best cat, *r*_1_ has random values in the range of [0,1], and *c*_1_ is a user-defined constant.
  (5) $$\begin{array}{c} \text{If }X_{\text{best},d}=1,\text{then }d_{\text{kd}}^{1}=-r_{1}c_{1},\text{ and }d_{\text{kd}}^{0}=r_{1}c_{1},\\ \text{If }X_{\text{best},d}=0,\text{then }d_{\text{kd}}^{1}=r_{1}c_{1}y,\;\;d_{\text{kd}}^{0}=-r_{1}c_{1}. \end{array}$$
Step 2: update values for *V*_kd_^1^ and *V*_kd_^0^ according to the expression, where *w* is the inertia weight and *M* is the number of columns.
  (6) $$\begin{array}{c} \begin{array}{cc} \begin{array}{c} V_{\text{kd}}^{1}=\omega V_{\text{kd}}^{1}+d_{\text{kd}}^{1},\\ V_{\text{kd}}^{0}=\omega V_{\text{kd}}^{0}+d_{\text{kd}}^{0}, \end{array} & d=1,\ldots ,M \end{array}. \end{array}$$
Step 3: calculate the velocity of cat_*k*_, *V*_kd_′, according to
  (7) $$\begin{array}{c} V_{\text{kd}}^{\prime }=\left\{\begin{array}{cc} V_{\text{kd}}^{1}, & \text{If }X_{\text{kd}}=0,\\ V_{\text{kd}}^{0}, & \text{If }X_{\text{kd}}=1. \end{array}\right. \end{array}$$
Step 4: calculate the probability of mutation in each dimension, defined by parameter *T*_kd_ which takes a value in the interval of [0,1]
  (8) $$\begin{array}{c} T_{\text{kd}}=\frac{1}{1+e^{-V_{\text{kd}}^{\prime }}}. \end{array}$$
Step 5: based on the value of *T*_kd_, the new value of each dimension of the cat is updated as follows:
  (9) $$\begin{array}{c} X_{\text{kd}}=\left\{\begin{array}{cc} X_{\text{best},d}, & \text{If rand}<t_{\text{kd}},\\ X_{\text{kd}}, & \text{If }t_{\text{kd}}<\text{rand}, \end{array}\right.\;d=1,\ldots ,M. \end{array}$$

The maximum velocity vector of *V*_kd_′ must be limited to value *V*_max_.

If the value of *V*_kd_′ surpasses that of *V*_max_, *V*_kd_′ must be selected for the corresponding velocity dimension.

**Figure 2:**
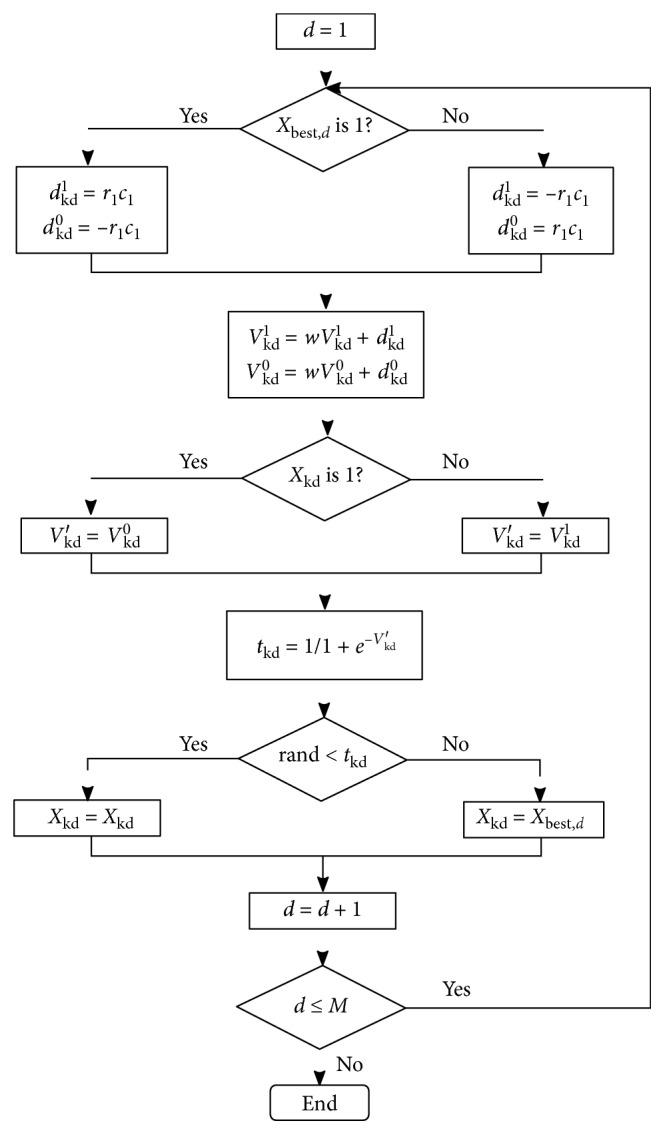
Tracing mode.

The following is a flow chart for a cat in the tracing mode (Figure 2).

## 5. Solving the Manufacturing Cell Design Problem (MCDP)

To solve the MCDP, it is essential to use a repair method for solutions that were not feasible.  Algorithm 2 describes the pseudocode used to solve the MCDP.

## 6. Repair Method

A solution may not satisfy the constraints, resulting in an unworkable solution. For this reason, the value that violates the constraint is repaired instead of the matrix being removed. In this section, a function is described to transform nonfeasible solutions into feasible solutions.

Thus, Algorithm 3 presents a repair method in which all rows not covered are identified and assigned accordingly. This will cover all restrictions.

## 7. Autonomous Search

Autonomous Search (AS) is a modern approach that allows the solver to automatically reconfigure its resolution parameters to provide better performance when bad results are detected [40].

In this context, performance is assessed through indicators that collect relevant information during the search. Search parameters are then updated advantageously according to the results obtained by the fitness evaluation.

This approach has been effectively applied to different optimization and satisfaction techniques, such as Constraint Programming [41], SAT [42], mixed integer programming [43, 44], and various other metaheuristic techniques [45–47].

In the present investigation, a version of the BCSO with Autonomous Search has been implemented, where the mixture ratio (MR) variable is used as an autonomous parameter; i.e., the MR value changes while the program is executed to give a more dynamic algorithm that directly influences the mode that the cat will take.

Algorithm 4 is the pseudocode describing the Autonomous Search BCSO.

## 8. Results

The BCSO implementation process of MCDP has led to results that will be presented in the following section. The metaheuristic was programmed in the JAVA programming language. For the execution of the algorithm, the parameters considered were the following:

- Iterations = 5000
- Number of cats = 30
- MR = 0.75 (75% seeking; 25% tracing)
- SMP = 15
- CDC = 0.2
- PMO = 0.76
- *w*=1
- *c*1=1
- *r*1 ∈ [0,1]

## 9. Boctor Instances

Tests with the implemented solution were carried out based on 90 instances of 16 × 30 matrices, obtained from 10 problems found in the paper of Boctor [48], hereafter called Boctor Instances. These problems included the use of 2 or 3 cells. In the case of 2 cells, the maximum number of machines (*M*_max_) in each took values between 8 and 12. In the case of 3, *M*_max_ varied between 6 and 9 machines per cell. In both cases, the value of *M*_max_ remained constant throughout the execution of the algorithm.

The values obtained by submitting each problem to the Classic BCSO and BCSO with Autonomous Search are summarized in Tables 1–9, where “*O*” denotes the global optimum given in [48]; “BCSO,” the best value obtained by the BCSO here proposed; “*A*,” the average number of optima obtained; “*I*,” the average number of iterations in which the optimum is reached; ”Ms,” the time (in milliseconds) used to reach the optimum; and “RPD,” the Relative Percent Difference, calculated as follows:

(10) $$\begin{array}{c} \text{RPD}=\frac{Z-Z_{\text{opt}}}{Z_{\text{opt}}}*\;100, \end{array}$$

where *Z*_opt_ is the best known optimal value and *Z* is the best optimal value achieved by BCSO.

The above results were run 40 times for each of the 90 Boctor Instances. It is important to point out that 100% of these were optimized, proving that BCSO can work with any MCDP instance. The performance of the BCSO metaheuristic in its Autonomous Search version was slightly better, demonstrated by some of the optima averages reached in the experimental results.

## 10. Other Author Instances

To analyze the effectiveness of the implemented algorithm in a wider range of problems, new instances from different authors were investigated. Matrix sizes ranged from 5 to 40 machines and from 7 to 100 parts.  Table 10 shows the instances used:

In order to improve the quality of the exhibited behavior by the autonomous version of the Binary Cat Swarm Optimization, we performed a detailed comparison by using these new instances, because they are hardest. This comparison includes two well-known metaheuristics: the first one is inspired by the behavior of the Egyptian vulture (EVOA) [71], and the second one mimics the flashing behavior of fireflies [72].  Table 11 reports the result comparison between our proposal and the methods published in [73].

If it observes the showed results for instances CF01 to CF11, we can conclude that BCSO presents a similar performance to EVOA. In both cases, the optimal values are reached. Moreover, we note the worst and mean values are equal. This behavior can be attributed to the similarity of the operations between both algorithms. Now, if it evaluates MFAO with respect to BCSO, we again can report a similar conclusion. Nevertheless, in CF05 and CF07, BCSO achieves two optimal values that they are not reached with MFAO.

From CF12 onwards, BCSO begins to exhibit an outstanding performance. For instance, in CF12, BCSO is the only one that finds the best solution (optimum value) reaching RPD 0%. Its closer competitor (MBFA) obtains RPD 28.57%. However, the biggest significant difference can be seen from CF15. In this instance, BCSO exhibits higher efficiency than EVOA and it overcomes the reached value by MBFA. Now, if taken any instances between CF16 and CF 35 (more than 57% of instances), the good yield of the BCSO exceeds the two compared approaches term of the best-found values, average-found values, and worst-found values also. Therefore, we can state that BCSO is more than a competitive technique. It is a real alternative for solving the Manufacturing Cell Design Problem.

Now, the values obtained by submitting each problem to Classic BCSO and BCSO with Autonomous Search are summarized in Table 12, where the global optimum is given in [74].

The above results were obtained after 40 executions for each of the 35 new instances. It should be noted that it was possible to reach optima in 100% of instances for both algorithms, proving that BCSO can work with almost any instance. The performance of the BSCO metaheuristic in its Autonomous Search version was slightly better, demonstrated in some of the optima achieved, improving by 3% with respect to the original.

## 11. Results for Boctor Instances Using BCSO and BCSO with Autonomous Search

**Figure 3:**
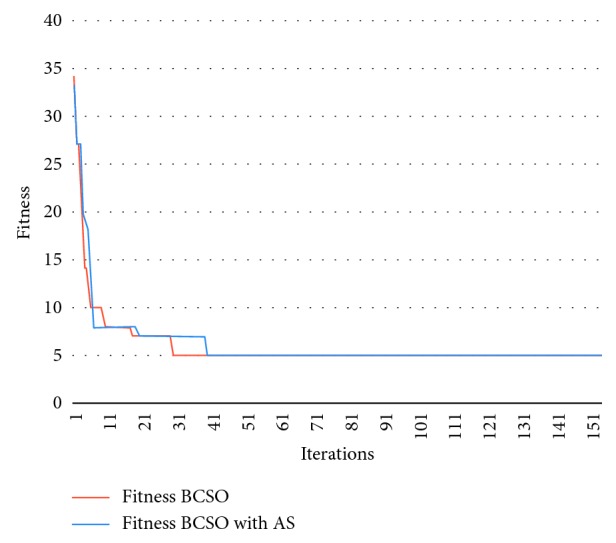
Graph showing the results of problem 7 for BCSO and BCSO AS with *C* = 3.

Figure 3 shows the results of the experiments conducted for the Boctor Instances presented above. Thanks to the operation mode of the BCSO, a fast optimum convergence is obtained at *C* = 2; however, when *C* = 3, the BCSO does not converge as quickly that said, the optimum is reached in most cases before 100 executions, which demonstrates the effectiveness of the proposed approach.

**Figure 4:**
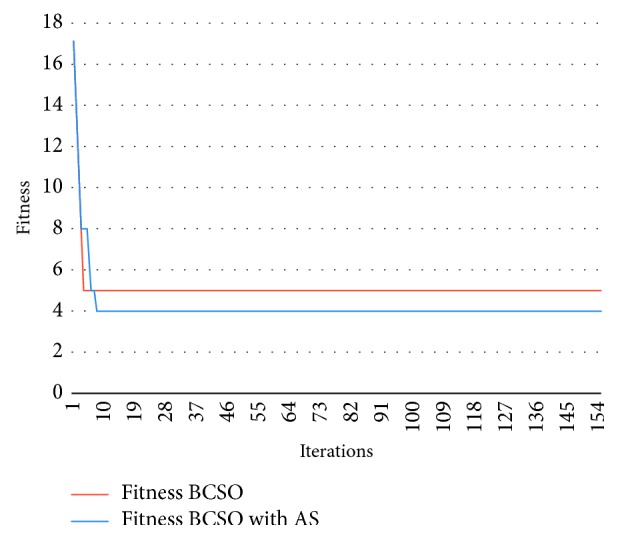
Graph showing the results of problem 3 for BCSO and BCSO AS with *C* = 2.

Figure 4 shows the results of problem 3, *C* = 2 and *M*_max_ = 8, over iterations. Both versions converge quickly: while the Autonomous Search BCSO reaches the optimum early (iteration 10), the normal BCSO is stuck at optimum of fitness 5 at iteration 4.

The following graph (Figure 3) shows the results of problem 7, with *C* = 3, *M*_max_ = 8, reaching the overall optimum in both cases at similar iterations: normal BCSO, iteration 30; and Autonomous Search BCSO, iteration 40.

## 12. Results for New Instances Using BCSO and BCSO with Autonomous Search

**Figure 5:**
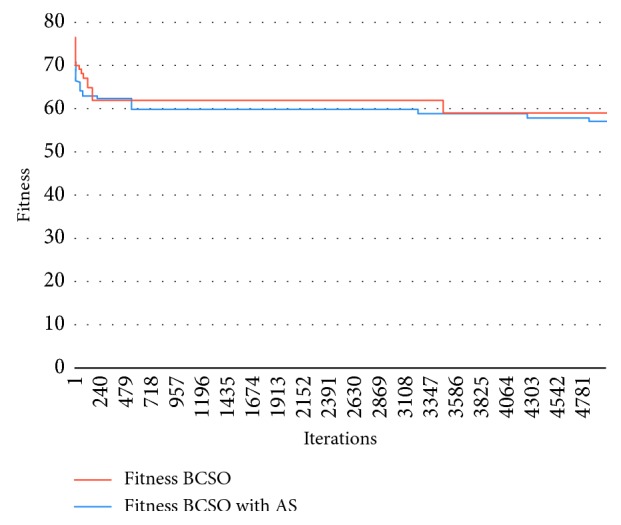
Graph showing the results of problem 26 for BCSO and BCSO AS.

Figure 5 shows the results of the experiments performed for new instances, in which it can be seen that the Autonomous Search algorithm helps the solution not to get trapped at some local optimum; however, not all results with Autonomous Search present an advantage over the original version.

Figure 5 represents the results of problem 26, with *M* = 24, *P*=40, *C* = 12, and *M*_max_ = 3, in which it can be seen that Autonomous Search BCSO does not have a great difference over the normal BCSO; however, Autonomous Search BCSO is able to explore new solutions, which makes it achieve better results.

**Figure 6:**
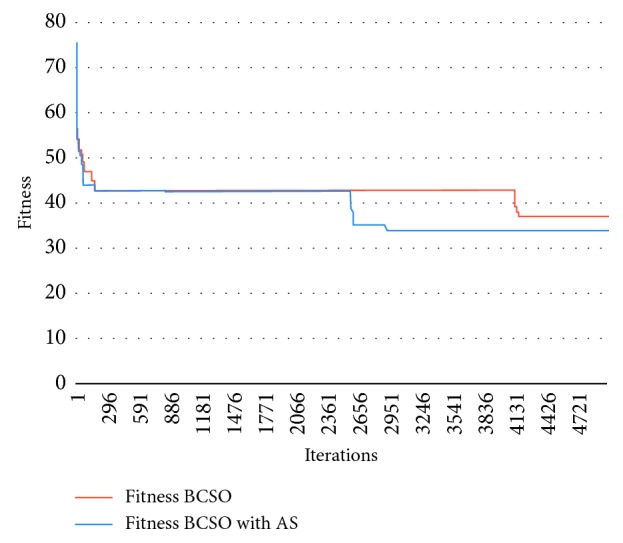
Graph showing the results of problem 30 for BCSO and BCSO AS.

The graph in Figure 6 represents the results of problem 30, with *M* = 30, *P*=41, *C* = 14, and *M*_MAX_ = 4, in which it can be seen that the Autonomous Search BCSO solutions continue to change without being trapped in a local optimum, whereas normal BCSO is trapped near iteration 4000.

**Figure 7:**
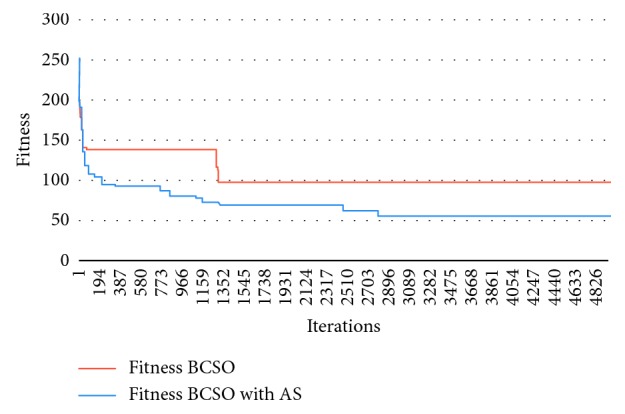
Graph showing the results of problem 35 for BCSO and BCSO AS.

The graph in Figure 7 represents the results of problem 35, with *M* = 40, *P*=100, *C* = 10, and *M*_max_ = 6, in which Autonomous Search BCSO solutions are changing, exploring new solutions, expanding their search space early on, before iteration 3000; normal BCSO is trapped in a local optimum near iteration 1000.

## 13. Conclusions

In the present investigation, a new algorithm inspired by cat behavior, called Cat Swarm Optimization, was presented in solving the Manufacturing Cell Design Problem, used for placement of machinery in a manufacturing plant.

The proposed BCSO was implemented and tested using 90 Boctor Instances plus 35 new instances, for a total of 125 instances: The BCSO managed to obtain 100% of known optima in the 90 Boctor Instances, achieving rapid convergence and reduced execution times. In the case of the 35 new instances, it was possible to obtain 100% of the 13 known optima. It should be noted that these results were obtained after a long testing process, where the different parameters of the algorithm were calibrated based on experimentation. For that reason, Autonomous Search was implemented as an optimization method to influence variables in real time, which resulted in dynamic MR that slightly improved results obtained: 3% compared to the original, with 100% of the known optima, both for the 90 Boctor Instances and the 35 new instances.

As can be seen from the results, this metaheuristic behaves well in all observed cases. This research demonstrates that BCSO is a valid alternative for solving the MCDP. The algorithm works well, regardless of the scale of the problem. However, solutions obtained could be improved by using different parameters for each set of instances.

The BCSO performance was significantly increased after selecting a good repair technique. However, relying on a repair method leads us not to recommend the use of this algorithm for other types of problems because it is far less efficient than other techniques for more complex problems.

For future research, a more extensible configuration could be developed to cover a wider set of problems. It would also be interesting to implement this technique in conjunction with other recent metaheuristics where limited work on Autonomous Search exists such as cuckoo search, firefly optimization, or bat algorithms [75]. Finally, hybridization with learning techniques is another interesting research line to pursue, where feedback gathered for the self-tune phase could be processed with machine learning in order to better track the complete solving process.

## Figures and Tables

**Algorithm 1:**
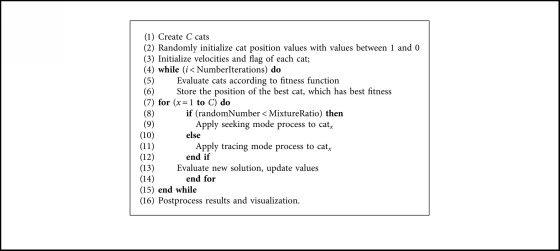
Binary Cat Swarm Algorithm.

**Algorithm 2:**
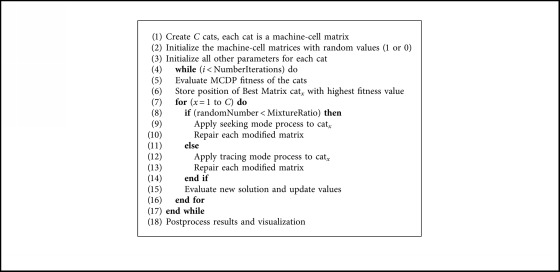
Solving MCDP.

**Algorithm 3:**
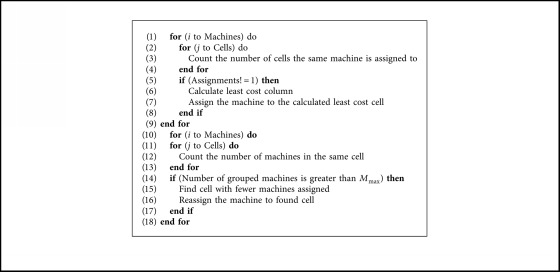
Repairing solutions.

**Algorithm 4:**
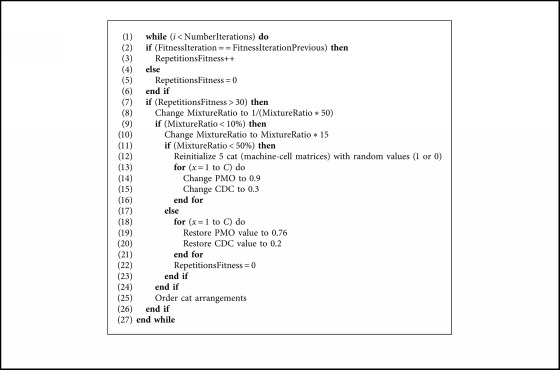
Autonomous search.

**Table 1:** Experimental results with cell = 2 and *M*_max_ = 8.

		Results for Boctor Instances with *C* = 2 *M*_max_ = 8									
*P*	*O*	Classic BCSO					Autonomous Search BCSO				
		BCSO	*A*	RPD	*I*	Ms	BCSO	*A*	RPD	*I*	Ms
1	11	11	11	0.00	5	30697.5	11	11	0.00	5	15977.6
2	7	7	7	0.00	7	27797.7	7	7	0.00	7	14487.6
3	4	4	4.05	0.00	79	27927.8	4	4	0.00	133	14175.7
4	14	14	14	0.00	5	29998.9	14	14	0.00	6	15149.6
5	9	9	9	0.00	93	28348.8	9	9	0.00	102	14358.0
6	5	5	5	0.00	5	28233.3	5	5	0.00	8	14165.0
7	7	7	7	0.00	5	28338.9	7	7	0.00	7	14562.7
8	13	13	13	0.00	6	28860.8	13	13	0.00	7	14895.6
9	8	8	8	0.00	6	28206.7	8	8	0.00	8	14583.8
10	8	8	8	0.00	19	28547.1	8	8	0.00	20	14750.2
$\bar{\text{X}}$	8.6	8.6	8.605	0.00	23	28695.7	8.6	8.6	0.00	30.3	14710.6

**Table 2:** Experimental results with cell = 2 and *M*_max_ = 9.

		Results for Boctor Instances with *C* = 2 *M*_max_ = 9									
*P*	*O*	Classic BCSO					Autonomous Search BCSO				
		BCSO	*A*	RPD	*I*	Ms	BCSO	*A*	RPD	*I*	Ms
1	11	11	11	0	5	19412	11	11	0	5	15109.4
2	6	6	6	0	5	17803.6	6	6	0	12	14284.1
3	4	4	4	0	5	16817	4	4	0	6	14140.3
4	13	13	13	0	5	18236.3	13	13	0	5	15027.4
5	6	6	6	0	81	16863.1	6	6	0	57	14228.9
6	3	3	3	0	10	16552.5	3	3	0	24	13997.8
7	4	4	4	0	5	17681.6	4	4	0	5	14432.9
8	10	10	10	0	7	18277.8	10	10	0	9	14734.4
9	8	8	8	0	5	17690.7	8	8	0	5	14473.7
10	5	5	5	0	7	18035.8	5	5	0	7	14645.4
$\bar{\text{X}}$	7	7	7	0	13.5	17737	7	7	0	13.5	14507.4

**Table 3:** Experimental results with cell = 2 and *M*_max_ = 10.

		Results for Boctor Instances with *C* = 2 *M*_max_ = 10									
*P*	*O*	Classic BCSO					Autonomous Search BCSO				
		BCSO	*A*	RPD	*I*	Ms	BCSO	*A*	RPD	*I*	Ms
1	11	11	11	0	5	18084.4	11	11	0	5	15047
2	4	4	4	0	9	16894.2	4	4	0	11	14336.8
3	4	4	4	0	5	16221.7	4	4	0	5	13991
4	13	13	13	0	6	17602.6	13	13	0	6	15051.8
5	6	6	6	0	7	16459.6	6	6	0	36	14620.4
6	3	3	3	0	8	16217.7	3	3	0	38	14853.1
7	4	4	4	0	5	16828.9	4	4	0	6	15199.7
8	8	8	8	0	8	17648.1	8	8	0	8	15858.6
9	8	8	8	0	6	16669.3	8	8	0	5	15226.4
10	5	5	5	0	6	16713.1	5	5	0	7	15591.5
$\bar{\text{X}}$	6.6	6.6	6.6	0	6.5	16933.96	6.6	6.6	0	12.7	14977.63

**Table 4:** Experimental results with cell = 2 and *M*_max_ = 11.

		Results for Boctor Instances with *C* = 2 *M*_max_ = 11									
*P*	*O*	Classic BCSO					Autonomous Search BCSO				
		BCSO	*A*	RPD	*I*	Ms	BCSO	*A*	RPD	*I*	Ms
1	11	11	11	0	5	17173.2	11	11	0	5	16818.2
2	3	3	3	0	7	16000.2	3	3	0	9	15159.5
3	3	3	3	0	8	15755.9	3	3	0	29	14537.3
4	13	13	13	0	6	17011.4	13	13	0	6	15634.2
5	5	5	5	0	9	16680.3	5	5	0	18	14958.3
6	3	3	3	0	6	16433.2	3	3	0	7	28722.4
7	4	4	4	0	5	16714.5	4	4	0	6	15837.8
8	5	5	5	0	6	17223.9	5	5	0	6	17155.2
9	5	5	5	0	10	16733.8	5	5	0	22	16827.5
10	5	5	5	0	6	16698.9	5	5	0	7	17077.1
$\bar{\text{X}}$	5.7	5.7	5.7	0	6.8	16642.53	5.7	5.7	0	12	17272.75

**Table 5:** Experimental results with cell = 2 and *M*_max_ = 12.

		Results for Boctor Instances with *C* = 2 *M*_max_ = 12									
*P*	*O*	Classic BCSO					Autonomous Search BCSO				
		BCSO	*A*	RPD	*I*	Ms	BCSO	*A*	RPD	*I*	Ms
1	11	11	11	0	5	18226.7	11	11	0	5	17694.2
2	3	3	3	0	6	16901.6	3	3	0	6	16481
3	1	1	1	0	8	16377.7	1	1	0	21	15203.2
4	13	13	13	0	5	17816.3	13	13	0	6	15927.9
5	4	4	4	0	6	16824.9	4	4	0	20	15004.5
6	2	2	2	0	35	16411.3	2	2	0	158	14512
7	4	4	4	0	6	16939	4	4	0	6	15269.6
8	5	5	5	0	7	17716.7	5	5	0	7	15861.7
9	5	5	5	0	8	17175.5	5	5	0	18	15091
10	5	5	5	0	6	20025.5	5	5	0	7	15258.2
$\bar{\text{X}}$	5.3	5.3	5.3	0	9.2	17441.52	5.3	5.3	0	25.4	15630.33

**Table 6:** Experimental results with cell = 3 and *M*_max_ = 6.

		Results for Boctor Instances with *C* = 3 *M*_max_ = 6									
*P*	*O*	Classic BCSO					Autonomous Search				
		BCSO	*A*	RPD	*I*	Ms	BCSO	*A*	RPD	*I*	Ms
1	27	27	27	0	40	23130.3	27	27	0	85	18900.6
2	7	7	7	0	12	21710.5	7	7	0	11	17891.1
3	9	9	9	0	38	21010.5	9	9	0	52	437225
4	27	27	27	0	10	22764.7	27	27	0	11	18664.8
5	11	11	11	0	11	21380.6	11	11	0	11	17956.1
6	6	6	6	0	13	20749.7	6	6	0	15	17323.4
7	11	11	11	0	60	21698.3	11	11	0	91	17904.1
8	14	14	14	0	14	22665.2	14	14	0	12	18360.5
9	12	12	12	0	12	21730.1	12	12	0	22	17725.5
10	10	10	10	0	27	22397.3	10	10	0	32	18011.2
$\bar{\text{X}}$	13	13.4	13	0	24	21923.7	13.4	13.4	0	34	59996.3

**Table 7:** Experimental results with cell = 3 and *M*_max_ = 7.

		Results for Boctor Instances with *C* = 3 *M*_max_ = 7									
*P*	*O*	Classic BCSO					Autonomous Search				
		BCSO	*A*	RPD	*I*	Ms	BCSO	*A*	RPD	*I*	Ms
1	18	18	18	0	42	22915.4	18	18	0	49	18995.1
2	6	6	6	0	14	21540.1	6	6	0	16	18216.9
3	4	4	4	0	27	20955.9	4	4	0	20	17177.4
4	18	18	18	0	21	23486.3	18	18	0	22	18684
5	8	8	8	0	15	19261	8	8	0	15	16942.8
6	4	4	4	0	28	18009	4	4	0	24	16508.8
7	5	5	5	0	35	18720	5	5	0	243	16994.5
8	11	11	11	0	14	300126	11	11	0	15	17666.8
9	12	12	12	0	16	21566.1	12	12	0	14	17191.1
10	8	8	8	0	16	21380.5	8	8	0	16	17549.1
$\bar{\text{X}}$	9	9.4	9	0	23	48796.1	9.4	9.4	0	43	17592.7

**Table 8:** Experimental results with cell = 3 and *M*_max_ = 8.

		Results for Boctor Instances with *C* = 3 *M*_max_ = 8									
*P*	*O*	Classic BCSO					Autonomous Search				
		BCSO	*A*	RPD	*I*	Ms	BCSO	*A*	RPD	*I*	Ms
1	11	11	11	0	16	21580	11	11	0	15	18090.8
2	6	6	6	0	20	20246.3	6	6	0	20	17017.4
3	4	4	4	0	17	19927.2	4	4	0	42	16878.1
4	14	14	14	0	19	21022.2	14	14	0	24	18642.2
5	8	8	8	0	36	19499.3	8	8	0	215	17156.4
6	4	4	4	0	31	19735.9	4	4	0	144	16542.7
7	5	5	5	0	30	20064.9	5	5	0	39	17352
8	11	11	11	0	19	21113.4	11	11	0	76	18459.6
9	8	8	8	0	36	20879.4	8	8	0	56	17950
10	8	8	8	0	17	19959.3	8	8	0	17	18353.6
$\bar{\text{X}}$	8	7.9	8	0	24	20402.8	7.9	7.9	0	65	17644.3

**Table 9:** Experimental results with cell = 3 and *M*_max_ = 9.

		Results for Boctor Instances with *C* = 3 *M*_max_ = 9									
*P*	*O*	Classic BCSO					Autonomous Search				
		BCSO	*A*	RPD	*I*	Ms	BCSO	*A*	RPD	*I*	Ms
1	11	11	11	0	13	21872.7	11	11	0	16	18462.7
2	6	6	6	0	20	20489.4	6	6	0	16	17624.9
3	4	4	4	0	14	20044	4	4	0	15	16748.8
4	13	13	13	0	15	22408	13	13	0	17	17698.7
5	6	6	6	0	69	22768.2	6	6	0	168	17120.3
6	3	3	3	0	69	19580.2	3	3	0	139	17167.1
7	4	4	4	0	24	20863.5	4	4	0	29	17602.5
8	10	10	10	0	66	23977.9	10	10	0	184	18202.8
9	8	8	8	0	15	26618.9	8	8	0	21	17849.1
10	5	5	5	0	17	20694.3	5	5	0	26	18483.6
$\bar{\text{X}}$	7	7	7	0	32	21931.7	7	7	0	63	17696.1

**Table 10:** New instances from other authors.

Problem	Author	Machines	Parts	Cells	*M*_max_
CFP01	King and Nakornchai [49]	5	7	2	3
CFP02	Waghodekar and Sahu [50]	5	7	2	4
CFP03	Seifoddini [51]	5	18	2	3
CFP04	Kusiak and Cho [52]	6	8	2	3
CFP05	Kusiak and Chow [53]	7	11	5	2
CFP06	Boctor [48]	7	11	4	2
CFP07	Seifoddini and Wolfe [54]	8	11	4	3
CFP08	Chandrasekharan and Rajagopalan [55]	8	20	3	4
CFP09	Chandrasekharan and Rajagopalan [56]	8	20	2	5
CFP10	Mosier and Taube [57]	10	10	5	4
CFP11	Chan and Milner [58]	10	15	3	4
CFP12	Askin and Subramanian [59]	14	24	7	3
CFP13	Stanfel [60]	14	24	7	3
CFP14	McCormick et al. [61]	16	24	8	5
CFP15	Srinivasan et al. [62]	16	30	6	6
CFP16	King [63]	16	43	8	4
CFP17	Carrie [64]	18	24	9	4
CFP18	Mosier and Taube [65]	20	20	6	7
CFP19	Kumar et al. [66]	23	20	7	6
CFP20	Carrie [64]	20	35	5	5
CFP21	Boe and Cheng [67]	20	35	5	5
CFP22	Chandrasekharan and Rajagopalan [68]	24	40	12	5
CFP23	Chandrasekharan and Rajagopalan [68]	24	40	7	5
CFP24	Chandrasekharan and Rajagopalan [68]	24	40	7	5
CFP25	Chandrasekharan and Rajagopalan [68]	24	40	11	5
CFP26	Chandrasekharan and Rajagopalan [68]	24	40	12	3
CFP27	Chandrasekharan and Rajagopalan [68]	24	40	12	3
CFP28	McCormick et al. [61]	27	27	6	11
CFP29	Carrie [64]	28	46	10	4
CFP30	Kumar and Vannelli [69]	30	41	14	4
CFP31	Stanfel [60]	30	50	13	3
CFP32	Stanfel [60]	30	50	14	4
CFP33	King-Nakornchai [49]	36	90	17	6
CFP34	McCormick et al. [61]	37	53	3	15
CFP35	Chandrasekharan and Rajagopalan [70]	40	100	10	6

**Table 11:** Comparison between classic BCSO.

ID	*M*	*P*	*C*	*M*_max_	Optimum values	EVOA				MBFA				CSOA			
						Best	Worst	Mean	RPD (%)	Best	Worst	Mean	RPD (%)	Best	Worst	Mean	RPD (%)
CF01	5	7	2	3	0	0	0	0	0.00	0	0	0	0.00	0	0	0	0.00
CF02	5	7	2	4	3	3	3	3	0.00	3	3	3	0.00	3	3	3	0.00
CF03	5	18	2	3	5	5	5	5	0.00	5	5	5	0.00	5	5	5	0.00
CF04	6	8	2	3	2	2	2	2	0.00	2	2	2	0.00	2	2	2	0.00
CF05	7	11	5	2	8	8	8	8	0.00	9	9	9	12.50	8	8	8	0.00
CF06	7	11	4	2	4	4	4	4	0.00	4	4	4	0.00	4	4	4	0.00
CF07	8	12	4	3	7	7	7	7	0.00	8	8	8	14.29	7	7	7	0.00
CF08	8	20	3	4	7	7	7	7	0.00	7	7	7	0.00	7	7	7	0.00
CF09	8	20	2	5	25	25	25	25	0.00	27	27	27	8.00	25	25	25	0.00
CF10	10	10	5	4	0	0	2	1.2	0.00	3	3	3	0.00	0	0	0	0.00
CF11	10	15	3	4	0	0	4	0.8	0.00	0	0	0	0.00	0	0	0	0.00
CF12	14	24	7	3	7	11	16	13.3	57.14	9	11	10.1	28.57	7	7	7	0.00
CF13	14	24	7	3	8	12	17	14.3	50.00	8	9	8.4	0.00	8	8	8	0.00
CF14	16	24	8	5	Unknown	30	35	32.9	—	36	41	39.6	—	24	24	24	—
CF15	16	30	6	6	Unknown	31	39	35.7	—	18	25	21.1	—	17	17	17	—
CF16	16	43	8	4	Unknown	42	47	44.6	—	39	46	43.8	—	29	30	29.05	—
CF17	18	24	9	4	Unknown	32	36	34.2	—	32	35	33.2	—	26	27	26.53	—
CF18	20	20	6	7	Unknown	46	53	49.9	—	52	59	56.2	—	41	42	41.18	—
CF19	20	23	7	6	Unknown	51	56	53.4	—	49	55	51.6	—	38	38	38	—
CF20	20	35	5	5	Unknown	28	42	36	—	7	16	12.3	—	2	2	2	—
CF21	20	35	5	5	Unknown	57	65	60.3	—	43	45	43.5	—	35		35	—
CF22	24	40	7	5	Unknown	30	43	37.5	—	0	23	15.5	—	0	5	4.9	—
CF23	24	40	7	5	Unknown	39	48	44.2	—	13	19	15	—	10	15	13.53	—
CF24	24	40	7	5	Unknown	44	53	49.7	—	25	30	27.6	—	18	22	20.98	—
CF25	24	40	11	5	Unknown	60	64	61.6	—	49	57	56.1	—	40	44	43.6	—
CF26	24	40	12	3	Unknown	68	71	70	—	64	67	65.6	—	59	63	62.15	—
CF27	24	40	12	3	Unknown	69	72	70.6	—	67	72	68.8	—	61	66	64.05	—
CF28	27	27	6	11	Unknown	84	100	94.1	—	76	97	92.1	—	54	54	54	—
CF29	28	46	10	4	Unknown	102	119	112.8	—	106	112	109.1	—	91	98	96.1	—
CF30	30	41	14	4	Unknown	57	63	59.7	—	43	65	58.3	—	37	43	42.6	—
CF31	30	50	13	3	Unknown	70	79	75.3	—	54	63	60.4	—	52	59	57.9	—
CF32	30	50	14	4	Unknown	86	90	87.6	—	76	81	77.6	—	66	75	72.15	—
CF33	36	90	17	6	Unknown	136	153	144.8	—	116	125	122.6	—	93	95	94.93	—
CF34	37	53	3	15	Unknown	352	383	369.2	—	325	335	329.5	—	256	256	256	—
CF35	40	100	10	6	Unknown	181	207	195.6	—	114	130	119.2	—	83	121	110.58	—

**Table 12:** Experimental results for new instances.

		Results with 35 new instances									
*P*	*O*	Classic BCSO					Autonomous Search BCSO				
		BCSO	*A*	RPD	*I*	Ms	BCSO	*A*	RPD	*I*	Ms
1	0	0	0	0	1	3583.2	0	0	0	1	3433.4
2	3	3	3	0	1	3619.7	3	3	0	1	3558.5
3	5	5	5	0	1	6273	5	5	0	1	6155.3
4	2	2	2	0	1	4157.7	2	2	0	1	3807.2
5	8	8	8	0	1	7897.8	8	8	0	1	7575.8
6	4	4	4	0	2	6919	4	4	0	2	6389.2
7	7	7	7	0	4	8158.6	7	7	0	5	7529.3
8	7	7	7	0	4	10048.4	7	7	0	4	9240.1
9	25	25	25	0	2	9373.3	25	25	0	2	8705.8
10	0	0	0	0	8	8982.7	0	0	0	10	8254.2
11	0	0	0	0	4	8893.5	0	0	0	4	8164.4
12	7	7	7	0	128	21747.6	7	7	0	194	19928.8
13	8	8	8	0	67	21835	8	8	0	87	20146.5
14	Unknown	24	24		103	27558	24	24		147	25383.5
15	Unknown	17	17		260	27283.5	17	17		296	25025.1
16	Unknown	29	29.05		922	40535.1	29	29.08		1268	37265.8
17	Unknown	26	26.53		717	31776.2	26	26.73		876	30591.1
18	Unknown	41	41.18		1241	26712.9	41	41.5		1174	27322.4
19	Unknown	38	38		577	31345.7	38	38.3		590	32086.5
20	Unknown	2	2		300	31251.8	2	2		358	29678.2
21	Unknown	35	35		318	34413.4	35	35.08		443	33892.7
22	Unknown	0	4.9		1909	42425.3	0	2.48		2017	103160.1
23	Unknown	10	13.53		1649	45014.9	10	12.43		1988	42954.3
24	Unknown	18	20.98		1958	45974.2	18	20.03		2080	43359.1
25	Unknown	40	43.6		2186	62062.9	40	44.08		2096	56834.7
26	Unknown	59	62.15		815	66630.4	57	60.73		2352	61659.3
27	Unknown	61	64.05		1204	66655.3	61	63.55		2202	62349.9
28	Unknown	54	54		466	43228.5	54	54.05		477	41759.4
29	Unknown	91	96.1		1434	76860.5	90	95.2		2578	67203.9
30	Unknown	37	42.6		1270	84810.9	34	40.9		2520	75800.3
31	Unknown	52	57.9		641	93391.4	49	54.3		2453	81183.7
32	Unknown	66	72.15		1670	99440.7	67	71.8		2431	87624.9
33	Unknown	93	94.93		2423	165907.8	93	95.48		1999	143833
34	Unknown	256	256		1345	70985.4	256	256		1005	70893.8
35	Unknown	83	110.58		964	153404.2	55	82.2		3579	150723
$\bar{\text{X}}$	5.85	34.51	36.63	0	703	42547.4	33.49	35.51	0	1007	41242.1

## Data Availability

The authors declare that the data used to support the findings of this study are available from the corresponding author.
